# Activity-dependent brain-derived neurotrophic factor expression regulates cortistatin-interneurons and sleep behavior

**DOI:** 10.1186/1756-6606-4-11

**Published:** 2011-03-09

**Authors:** Keri Martinowich, Robert J Schloesser, Dennisse V Jimenez, Daniel R Weinberger, Bai Lu

**Affiliations:** 1 Genes, Cognition and Psychosis Program (GCAP), National Institute of Mental Health (NIMH), Bethesda, MD 20892, USA; 2 Laboratory of Molecular Pathophysiology, National Institute of Mental Health (NIMH), Bethesda, MD 20892, USA; 3 Glaxo Smith Kline, Pudong, Shanghai, China

## Abstract

**Background:**

Sleep homeostasis is characterized by a positive correlation between sleep length and intensity with the duration of the prior waking period. A causal role for brain-derived neurotrophic factor (BDNF) in sleep homeostasis has been suggested, but the underlying mechanisms remain unclear. Cortistatin, a neuropeptide expressed primarily in a subset of cortical GABAergic interneurons, is another molecule implicated in sleep homeostasis.

**Results:**

We confirmed that sleep deprivation leads to an increase in cortical cortistatin mRNA expression. Disruption of activity-dependent BDNF expression in a genetically modified mouse line impairs both baseline levels of cortistatin mRNA as well as its levels following sleep deprivation. Disruption of activity-dependent BDNF also leads to a decrease in sleep time during the active (dark) phase.

**Conclusion:**

Our studies suggest that regulation of cortistatin-expressing interneurons by activity-dependent BDNF expression may contribute to regulation of sleep behavior.

## Background

Sleep behavior is dependent on two processes; circadian regulation as well as homeostatic regulation [1,2]. Circadian regulation dictates the distribution of sleep and waking over the 24-h cycle, while homeostatic regulation tracks sleep need [3]. Sleep pressure is increased by sleep deprivation (SD) and reduced by increased sleeping [4,5]. It is believed that slow wave activity (SWA) measured in the delta range of the electroencephalogram (EEG) (1.0-4.0 Hz) is regulated homeostatically, and it is hypothetically associated with synaptic plasticity [6,7]. Little is known about the biological processes responsible for sleep homeostasis - the sleep need as a function of previous wakefulness.

A recent study provided a biological link between synaptic plasticity in the cerebral cortex and sleep homeostasis [8]. There is a positive correlation between exploratory behavior during wakefulness, the induction of plasticity related genes including *BDNF*, *Arc*, *Homer *and *NGFI-A *in the cerebral cortex, and the extent of SWA, a sensitive marker for sleep pressure and sleep need [9]. A key follow-up study provided evidence that the degree of brain-derived neurotrophic factor (BDNF) expression during wakefulness is causally linked to the extent of SWA in the subsequent rest period [10].

BDNF is widely expressed in the developing and mature brain, and plays an important role in neuronal survival and differentiation during development, and in synaptic plasticity in the adult brain [11-13]. Both its gene transcription and its secretion are strongly regulated by neuronal activity [11,14,15]. During development, BDNF facilitates maturation of cortical inhibition and promotes the mature GABAergic phenotype [16-22]. In particular, fast-spiking parvalbumin-positive interneurons, which highly express the cognate receptor for BDNF, TrkB, are especially sensitive to BDNF signaling [16,23,24].

Cells expressing the neuropeptide cortistatin define a subset of GABAergic interneurons, which are found in highest abundance in the cerebral cortex and hippocampus [25,26]. Cortistatin expression is rapidly upregulated in the second week of rodent postnatal life [25]. Cortistatin-expressing cells partially co-localize with cells expressing somatostatin, parvalbumin and calbindin [25]. Previous studies showed that cortistatin may be preferentially localized to neurons that are double-positive for calbindin and parvalbumin [25]. Cortistatin is structurally related to somatostatin, but has biological functions that render it functionally distinct [27,28]. For example, in contrast to somatostatin, cortistatin enhances SWA and can antagonize the effects of cholinergic signaling on cortical excitability [28-30]. These previous studies have defined cortistatin as one of few molecules known to influence and correlate with sleep need.

In the present study, we show that a genetic manipulation that leads to disruption of activity-dependent BDNF expression results in impairments in sleep regulation and behavior that are consistent with a deficit in sleep homeostasis. In parallel, we show that this genetic alteration leads to misregulation of cortistatin gene expression. Our results suggest that BDNF regulation of sleep homeostasis may, at least in part, be mediated via activity-dependent BDNF regulation of a subpopulation of cortistatin-expressing interneurons.

## Results

### Basal levels of *Bdnf *expression in resting condition and after induced neuronal activity in BDNF-KIV animals

**Figure 1:**
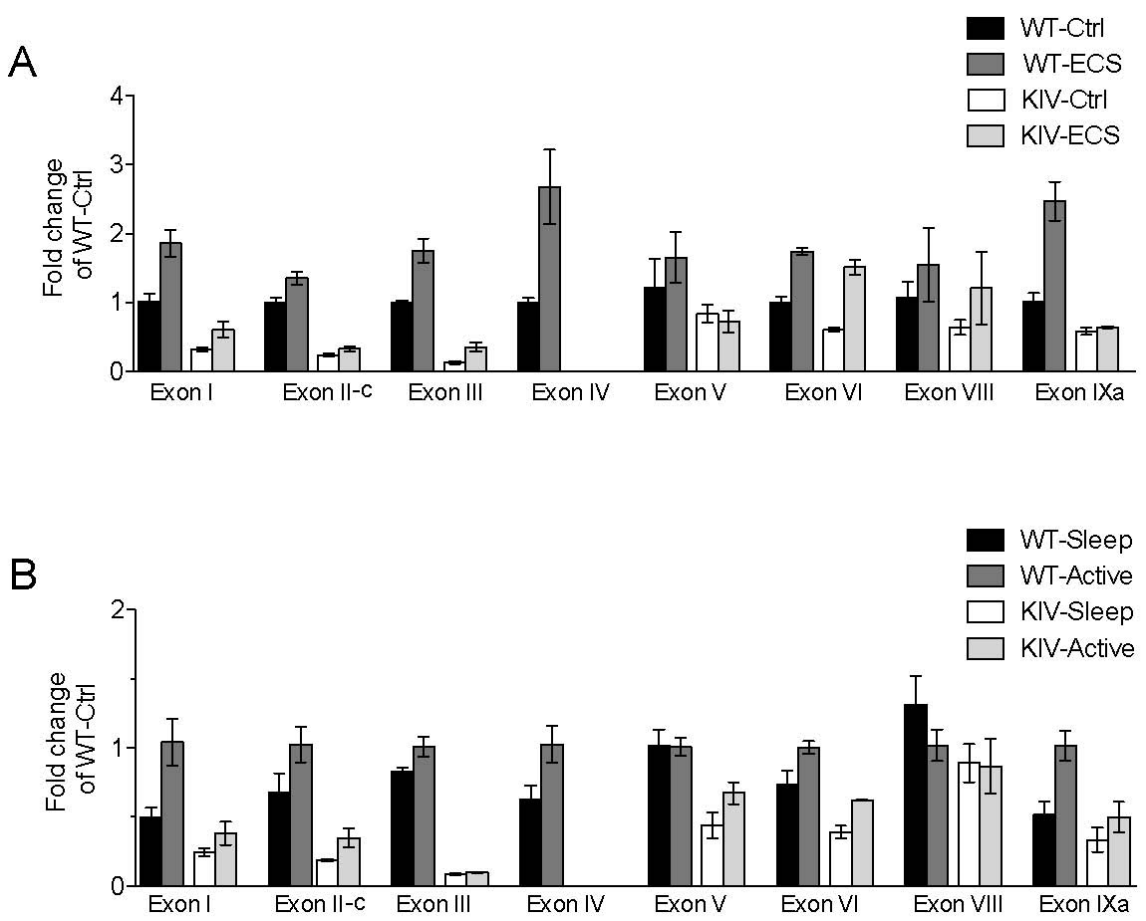
**Effect of neuronal activity on *Bdnf *transcripts**. All data are presented as fold changes over level of WT-Ctrl for that exon. (A). Relative expression levels of individual *Bdnf *transcripts at baseline (Ctrl) and after electroconvulsive shock (ECS) in the medial prefrontal cortex (mPFC) of WT and BDNF-KIV mice (n = 3 each condition). (B). Relative levels of individual *Bdnf *transcripts at the end of the lights on, rest cycle (Sleep) and at the end of the lights off, active cycle (Active) in WT and BDNF-KIV animals (n = 3 each group).

*Bdnf *gene transcription is driven by at least nine different promoters, each of which drives transcription of a short, 5' non-coding exon, which is spliced to a 3' common coding exon [31]. We have previously generated a knock-in transgenic mouse line where transcription of *Bdnf *from promoter IV, the promoter most sensitive to neuronal activity [32,33], is disrupted by inserting a green fluorescent protein (GFP)-STOP cassette into the *Bdnf *promoter IV locus (BDNF-KIV) [32]. In these mice, transcriptional activity from promoter IV leads to production of GFP in lieu of BDNF. We have previously shown that the activity-dependent expression of BDNF protein is nearly abolished in these mice [32]. The aim of the present study was to determine whether activity-dependent BDNF expression influences sleep behavior. We first analyzed *Bdnf *mRNA in the medial prefrontal cortex (mPFC) in the BDNF-KIV and their wild type (WT) littermates in regular, resting housing conditions without any manipulation. Surprisingly, we found that in addition to complete disruption of promoter IV-driven transcription, there was a significant dampening of basal *Bdnf *transcription, including transcription driven by promoters I, II, III, VI, and IXa in the resting condition, whereas basal transcription driven by promoters V and VIII was less affected (Figure 1A, WT-Ctrl versus KIV-Ctrl). For measurement of promoter II activity we analyzed transcript levels of the II-c variant. Since the II-a and II-b transcripts are driven by the same promoter, analyses of levels of these transcripts were nearly identical to the results obtained for the II-c variant except that their expression levels were significantly lower (data not shown). The observed dampening of transcription from alternative promoters likely results from promoter interference by the phosphoglycerate kinase (PGK) promoter. This PGK promoter was left intact in the mutant genome to drive expression of a neomycin selection cassette [32].

To confirm that activity-dependent *Bdnf *transcription is truly disrupted in the BDNF-KIV mice despite dampening of basal *Bdnf *expression, we measured levels of individual *Bdnf *transcripts in response to electroconvulsive shock (ECS), a condition in which neuronal activity is dramatically enhanced. As expected, in the WT mPFC, transcription driven by promoter IV was increased by the largest magnitude, while more moderate induction was seen from promoters I, II, III, VI and IXa (Figure 1A). In our experimental setup, levels of exon VII were so low that accurate quantification was not possible (data not shown). We further confirmed that no exon IV transcript could be detected in the mutant animals either before or after ECS (Figure 1A, KI-Ctrl versus KI-ECS, exon IV). These results, together with previous findings of relatively unaltered basal BDNF protein levels in the cortex of BDNF-KIV [32], suggest that the BDNF-KIV line remains an excellent tool to study the functional consequences of activity-dependent BDNF expression, although it cannot be claimed as promoter-IV specific.

### Circadian regulation of *Bdnf *expression and effect of sleep deprivation

Expression of *BDNF *has been shown to correlate with sleep need in the rat where its levels rise in correlation with the extent of wakefulness over the circadian day [34]. To determine whether and to what extent activity-dependent transcription contributes to circadian regulation of *Bdnf *expression, we compared transcript levels at the end of the active (dark, awake) period and at the end of the rest (light, sleep) period in WT and BDNF-KIV mPFC. Three observations were made. First, *Bdnf *transcription from promoters I, II, III, IV and IXa increased during wakefulness in the WT animals (Figure 1B, WT-Sleep versus WT-active). Second, there was a decrease in levels of exon I, II-c, III, V, VI and IXa transcripts at the end of the rest cycle (sleep) in the mutant animals (Figure 1B, WT-Sleep versus KIV-sleep). These data are almost identical to those seen in our previous experiment under resting conditions (Figure 1A, except promoter V), suggesting similar interference mechanisms by the PGK promoter are operative. Third, in addition to a complete blockade of transcription induction from promoter IV over the waking cycle, the difference in *Bdnf *transcript levels between WT and BDNF-KIV animals during the waking cycle was magnified from promoters I and IXa (Figure 1B and Additional File 1, table S1).

**Figure 2:**
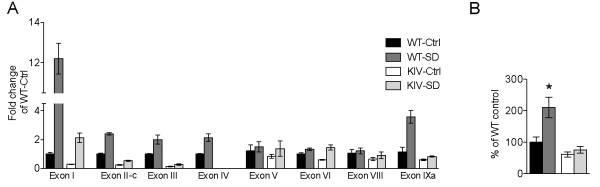
**Effect of sleep deprivation on *Bdnf *transcripts and protein levels**. (A). Relative levels of individual *Bdnf *transcripts at baseline (Ctrl) and after 12 h of sleep deprivation (SD) in WT and BDNF-KIV animals. Data are quantified and presented as in Figure 1 (n = 3 each condition). (B). Levels of total BDNF protein at baseline (Ctrl) and after 12 h of sleep deprivation (SD) in WT and BDNF-KIV animals. Data is presented at % induction over WT-Ctrl (n = 4 each group).

The molecular and cellular effects that occur over the course of a circadian waking session can be magnified when animals are exposed to sleep deprivation (SD). To gain insight into the role of individual *Bdnf *transcripts in sleep behavior, we performed a similar transcript analysis in WT and BDNF-KIV animals after exposure to 12-hours of SD. Transcription from promoters I, II, III, IV and IXa was increased in the WT mPFC, with promoter I showing the most robust effect as demonstrated by a 12-fold induction (Figure 2A, WT-Ctrl versus WT-SD, exons I, II-c, III, IV and IXa). In addition, for transcripts I and IXa the SD-induced increase in transcription was significantly attenuated in the BDNFK-IV animals (Figure 2A and Additional File 1, table S1), suggesting that the magnitude of *Bdnf *induction from multiple promoters following SD is impaired in BDNF-KIV when compared to WT mice (Figure 2A). This suggests that in conjunction with the complete loss of promoter-IV driven increases, the induction of *Bdnf *transcription in response to SD is attenuated in the BDNF-KIV mice.

We followed up these experiments by determining the effects of SD on BDNF protein expression in WT and mutant animals. In agreement with our mRNA results we found that there was a moderate decrease in the baseline levels of BDNF protein expression in the mutant animals, but that the induction of BDNF expression following SD was highly impaired in the mutant animals (Figure 2B). Consistent with this result, our previous study demonstrated that the increase in BDNF protein induced by high-K^+ ^in embryo-derived cultured cortical neurons and that the *in vivo *increase in BDNF protein induced by kainic acid in the frontal cortex was completely abolished in BDNF-KIV mice [32]. Thus, the impact of the PGK promoter on downregulation of transcription from alternative *Bdnf *promoters may contribute to the complete lack of BDNF protein induction in response to multiple neuronal-activity manipulations. These data, in conjunction with our previous studies, suggest that the BDNF-KIV line is an ideal model to study the impact of loss of activity-dependent BDNF expression.

**Figure 3:**
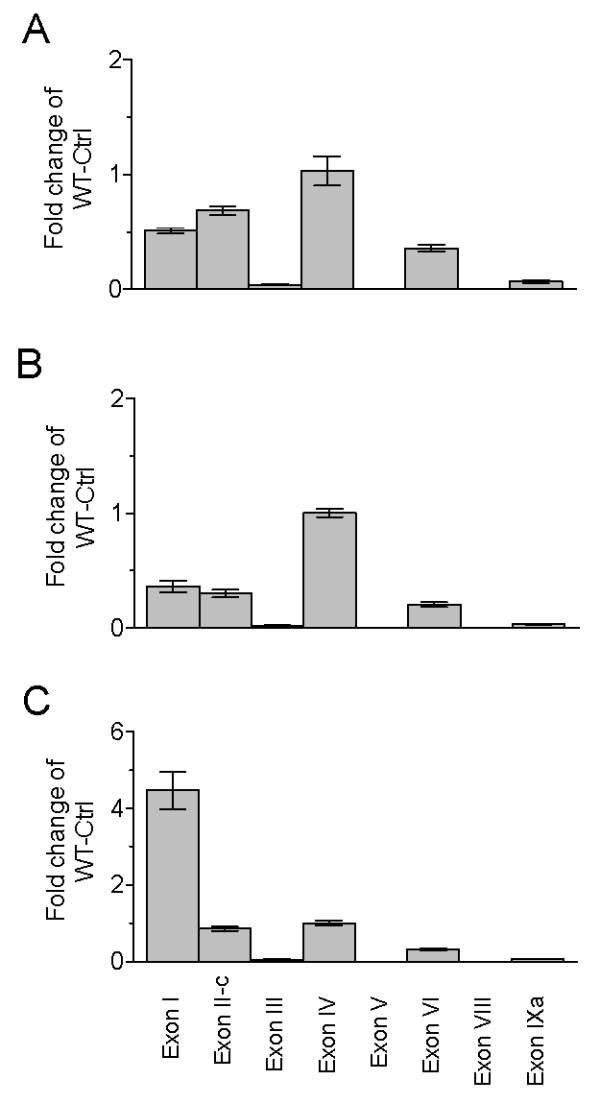
**Relative abundance of individual *Bdnf *transcripts at baseline, after ECS and after SD**. Relative levels of individual *Bdnf *transcripts in the mPFC of WT animals are reported at baseline (A), after ECS (B) and after 12 h SD (C). Data in A, B and C are presented as the fold change relative to levels of exon IV transcript levels in each individual experiment (n = 4 each group).

The genetic misregulation in these mutant animals is fairly complex with disruption of all promoter IV-driven activity as well as significant down-regulation from alternative promoters both at baseline and in response to different conditions of increased neuronal activity. Therefore, to better interpret our data, we sought to understand the abundance of individual transcripts in these different environmental conditions. A quantitative analysis was conducted to determine the relative abundance of individual *Bdnf *transcripts in WT animals under baseline conditions (Figure 3A), after ECS (Figure 3B) and after SD (Figure 3C). As expected, we found that under baseline conditions and after ECS, exon IV-containing transcripts were the most abundant in the frontal cortex (Figure 3A, 3B). In contrast, after 12-hour SD, exon I-containing transcripts overtook exon IV-containing transcripts to become the most abundant (Figure 3C).

### Dysregulation of *cortistatin *gene expression

**Figure 4:**
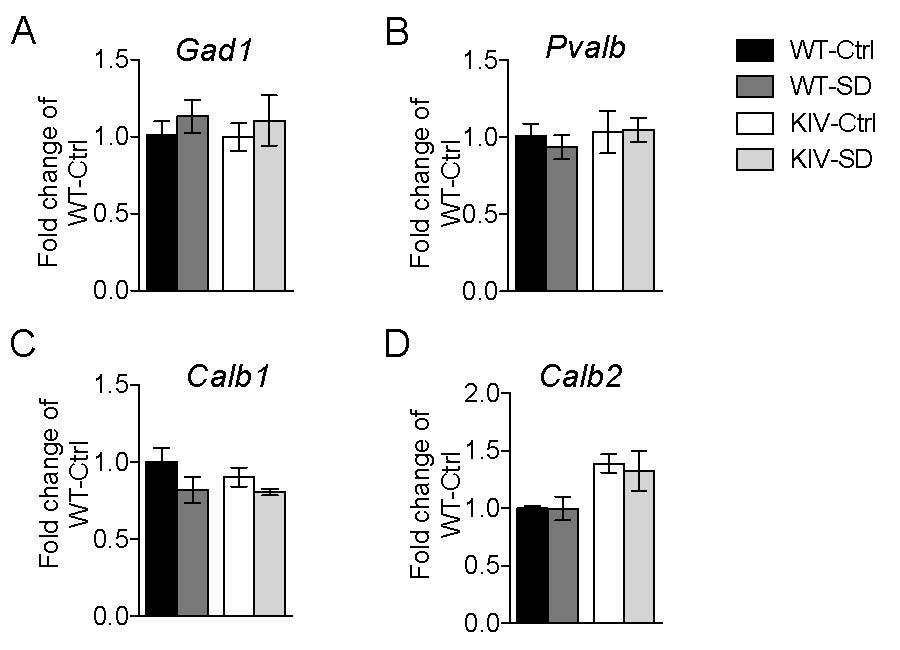
**Normal expression of GABAergic markers in BDNF-KIV mice**. Relative mRNA levels of markers of GABAergic interneurons in mPFC of WT and BDNF-KIV were measured at baseline (Ctrl) and after 12 h sleep deprivation (SD). Data is presented as fold change over WT Ctrl for *Gad1 *(A), *Pvalb *(B), *Calb1 *(C) and *Calb2 *(D) (n = 3 each group).

The BDNF-KIV mice exhibit deficits in cortical inhibitory transmission and a small, but significant reduction in parvalbumin immunofluoresence in the mPFC [32]. We asked whether activity-dependent BDNF signaling may influence the expression of the neuropeptide cortistatin, which is expressed in a subset of cortical GABAergic interneurons and has been implicated as a molecule that correlates with sleep need and influences sleep homeostasis [25,27,35]. We first examined the mRNA expression levels of markers of inhibitory interneurons including *Gad1*, as well as the genes that encode the calcium-binding proteins parvalbumin, calbindin and calretinin (*Pvalb, Calb1 and Calb2*, respectively). There was no significant change in the mRNA expression levels of these markers between WT and mutant animals either at baseline or following SD (Figure 4A, 4B, 4C and 4D).

**Figure 5:**
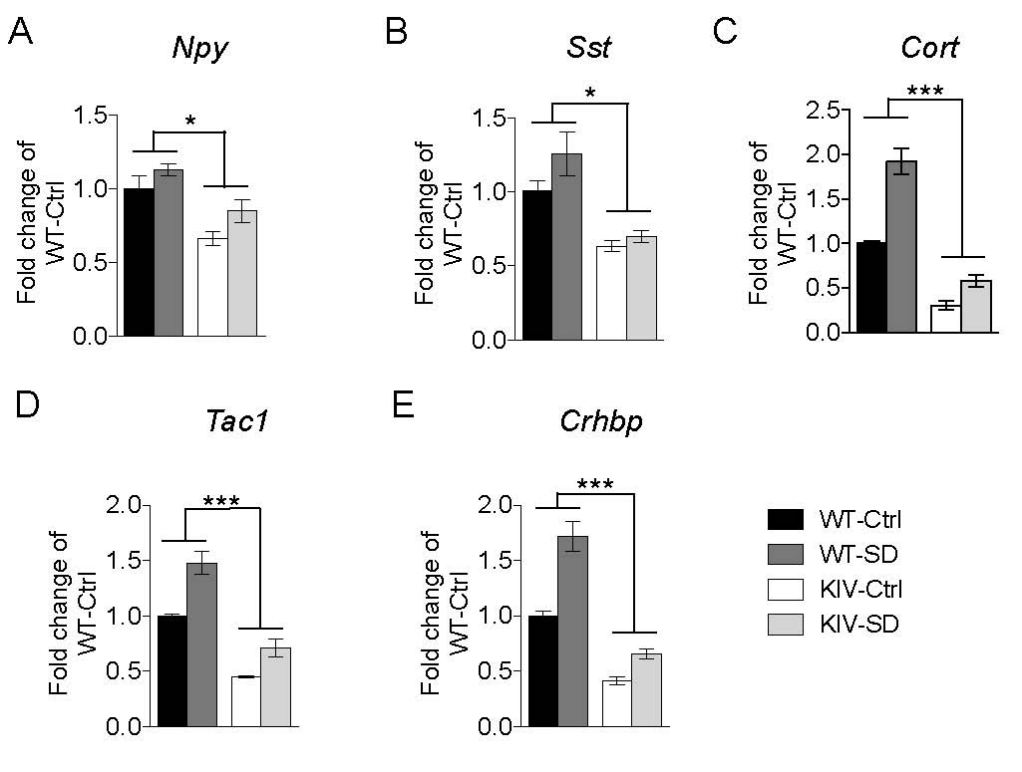
**Dysregulation of neuropeptide genes in subsets of GABAergic interneurons**. Relative levels of neuropeptides in a subset of GABAergic interneurons at baseline (Ctrl) and after 12 h sleep deprivation (SD) in WT and BDNF-KIV animals. Data is presented as fold change over WT Ctrl for *Npy *(A), *Sst *(B), *Cort *(C), *Tac1 *(D) and *Crhbp *(E) (n = 3 each group).

Next we analyzed markers of several neuropeptides, which have been shown to co-localize with, as well as to define, specific sub-populations of cortical interneurons. We found differences in the mRNA levels of the genes encoding neuropeptide Y, somatostatin, cortistatin, substance P and corticotropin-releasing hormone binding-protein (*Npy, Sst, Cort, Tac1 and Crhbp*, respectively), with the most striking down-regulation in *Cort *expression (Figure 5A, 5B, 5C, 5D and 5E). Interestingly, *Cort, Tac1 *and *Crhbp *show significant increases in expression levels in response to SD (Figure 5C, 5D, 5E). Thus, these results may characterize a subpopulation of cortical interneurons that is particularly sensitive to activity-dependent BDNF signaling and play a role in regulation of sleep homeostasis.

### Disruption of activity-dependent BDNF expression leads to changes in sleep behavior

**Figure 6:**
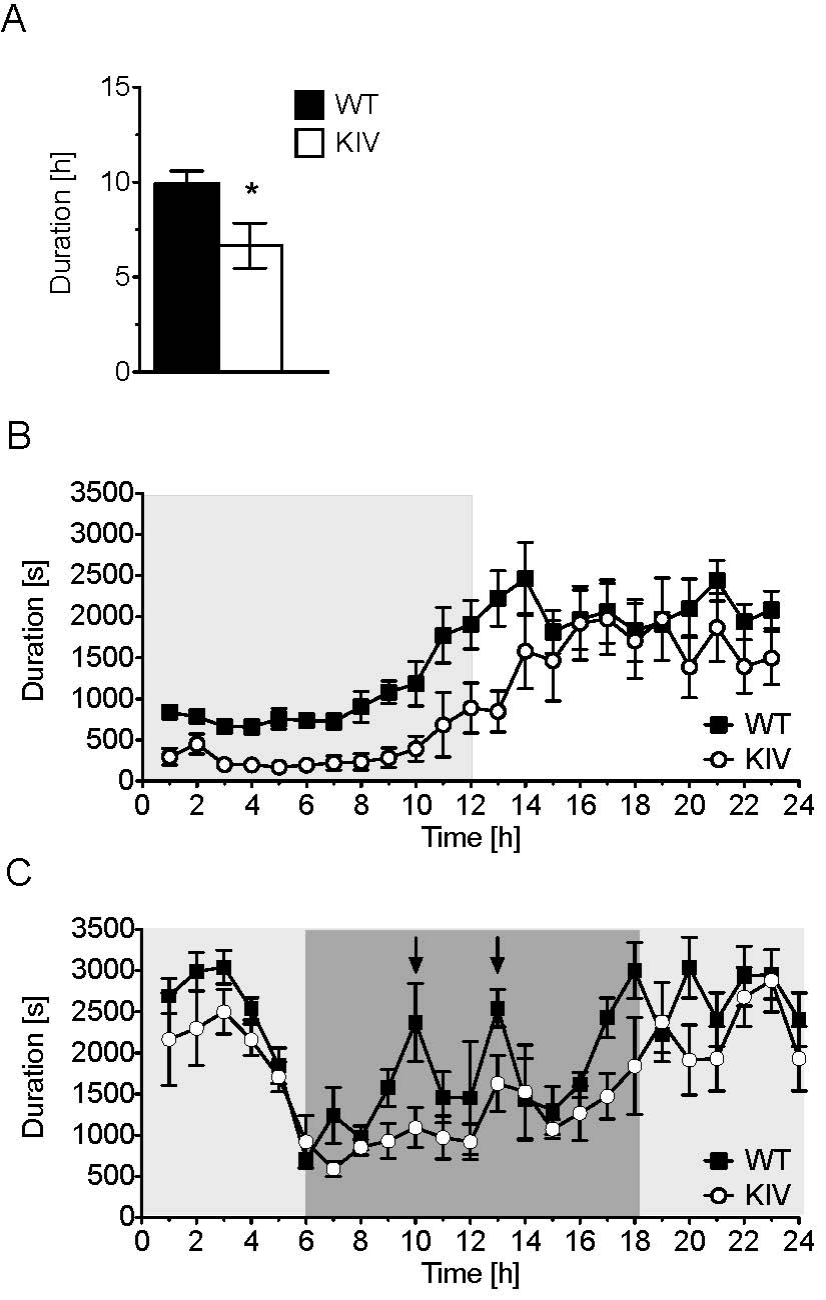
**Changes in sleep behavior due to disruption of activity-dependent BDNF expression**. (A). Total time over a 24 h session spent sleeping by WT and BDNF KIV mice (n = 8 each genotype). (B). Hour-by-hour plot of sleep behavior over the 24 h period with lights off phase (1-12 h) indicated by shading. (C). Hour-by-hour plot of sleep behavior under conditions of constant darkness. Dark shaded area represents period of time corresponding to animals' original lights off, active phase. Arrows point to conserved rest periods during the active phase in WT animals (n = 4 each genotype).

To determine the effect of blockade of activity-dependent BDNF expression on sleep behavior, we used automated home-cage monitoring to analyze the sleep-wake cycle. BDNF-KIV animals exhibited a substantial decrease in the total amount of time spent sleeping over a complete 24 h period compared to WT animals (Figure 6A). Analysis of an hour-by-hour activity plot revealed that the decrease in total sleep time resulted from less time spent sleeping during the active (dark) phase as well as a substantial delay before mutant animals commence their rest cycle (Figure 6B).

Several previous studies using C57Bl/6J mice have reported discrete breaks of inactivity in circadian wheel running during the dark (active) period [36-39]. These data have contributed to the idea that mice take a nocturnal nap during their active phase, a phenomena coined "siesta sleeping", as a means to discharge accumulated sleep pressure [40-42]. Consistent with these reports, we were able to detect this "siesta sleep" in WT mice with home cage monitoring when animals were observed under conditions of constant darkness. Under constant darkness conditions, two distinct peaks of sleep behavior during the active phase were revealed in WT animals. However, no such peaks were observed in the BDNF-KIV animals (Figure 6C). These results raise the possibility that activity-dependent BDNF expression may also be important in regulation of "siesta sleep".

## Discussion

We report here that BDNF-KIV mice slept less overall than WT mice. This decrease was accounted for by less sleep in the later parts of the active (dark) cycle and a delay in entering the rest phase. With progression of the active cycle or in response to SD, both *Bdnf *and *Cort *expression increase as sleep pressure builds. Consistent with this notion, we observed a rise in both *Bdnf *and *Cort *gene expression in WT animals after exposure to SD. However, in BDNF-KIV animals the levels of BDNF mRNA and protein as well as *Cort *gene expression were impaired. The genetic manipulation in the BDNF-KIV animals results in complete disruption of promoter IV-derived BDNF and down-regulation of additional *Bdnf *transcripts, resulting in a complete loss of the SD-induced increase in BDNF protein expression. Interestingly, transcription driven by promoter I was the most highly regulated in response to SD. In comparison to the 2-fold increase in promoter IV-driven transcription, promoter I driven transcription was elevated 12-fold. This difference is quite striking compared to the effects of ECS, which enhanced promoter IV activity by ~3-fold and promoter I activity by ~2-fold. Indeed, following SD, exon I-containing transcripts became the most highly expressed in the mPFC, with exon I-containing transcripts being expressed 4-fold more abundantly than exon IV-containing transcripts. Since the large SD-induced increase in exon I-driven transcription is significantly attenuated in addition to the complete disruption of promoter IV-driven activity, the BDNF-KIV mutant animals provide a very useful tool for studying the effects of activity-dependent BDNF expression on sleep homeostasis and behavior.

BDNF has been implicated in the development and function of the cortical inhibitory system at multiple levels [16,43-45]. Several studies have suggested that BDNF plays an important role in regulating inhibitory interneuron migration in the cerebral cortex [46-48]. BDNF also plays a key role in inhibitory interneuron differentiation and maintenance later in neurodevelopment [16,43,45]. Substantial evidence has shown that BDNF signaling through TrkB is critical in promoting inhibitory interneuron synaptogenesis and development of a mature GABAergic phenotype by inducing the expression of markers of GABAergic cells including GAD67, GAT1, calcium-binding proteins including parvalbumin as well as various neuropeptides [16,19,22,43,45]. Functionally, the impact of BDNF signaling on promoting maturation of cortical inhibition results in regulation of the critical period for plasticity in the visual cortex [16]. The onset of the critical period for visual development coincides with the initiation of non-REM sleep homeostasis, and monocular deprivation experiments have shown that sleep enhances synaptic remodeling during the critical period of visual cortex development [49]. It is of interest that the developmental induction of cortistatin expression at the end of the second postnatal week in rodent life coincides with rapidly rising cortical BDNF levels, initiation of the critical period for visual cortical plasticity, and onset of non-REM sleep [16,25,49-51]. The present study demonstrates a role for activity-dependent BDNF expression in regulation of sleep behavior, possibly via effects on a subset of cortical GABAergic interneurons. These results may have implications in critical period plasticity in visual cortex as well as other cortical areas.

In the adult brain cortistatin expression is correlated with sleep need and its administration promotes slow-wave activity (SWA) [27,35]. *Cort *gene expression is highest at the end of the circadian active period and increases dramatically in response to SD [27,35]. Functionally, it has been shown that intracerebroventricular administration of cortistatin leads to induction of SWA [27,35]. Interestingly, the increase in SWA observed after SD only occurs in rats after postnatal day 20, coinciding with the time when SD begins to induce cortical BDNF expression [52]. This time period also coincides with electrophysiological maturation of the inhibitory interneuron system and attainment of maximal slow wave delta power [50,53].

It has been reported that enhancing BDNF expression via exploratory activity during waking is causally linked to homeostatic sleep mechanisms [9,10]. Some studies have suggested that BDNF protein, which increases during sustained waking, leads to heightened synaptic potentiation and increased cell-to-cell coupling [6,7,9,10]. This in turn, leads to increased cortical synchrony and, subsequently, increased SWA power [6,7,9,10]. Our results suggest that a subset of GABAergic interneurons expressing several neuropeptide markers, including cortistatin, are highly sensitive to activity-dependent BDNF signaling and sleep pressure. Expression of *Crhbp*, a binding protein that acts to sequester corticotropin releasing hormone (CRH) may also define this subset of interneurons. *Crhbp *expression is also misregulated in the BDNF-KIV mice, is induced following sleep deprivation, and has been co-localized with NPY-positive interneurons [54]. Recent studies have shown that CRH may also be capable of modulating sleep homeostasis [55]. Future experiments should directly examine whether regulation of sleep homeostasis by activity-dependent BDNF expression is mediated by this subpopulation of cortistatin-positive GABAergic interneurons.

## Conclusions

In conclusion, we have shown that a genetic manipulation that disrupts activity-dependent BDNF expression results in behavioral impairments that are consistent with a deficit in sleep homeostasis. We also show that this genetic alteration leads to a substantial misregulation of *Cort *gene expression. Our results raise the possibility that BDNF regulation of sleep behavior may, at least in part, be mediated via activity-dependent BDNF regulation of cortistatin-positive interneurons.

## Methods

### Animals

BDNF-KIV animals were generated as described previously [32], and further backcrossed onto a pure C57Bl/6 background. Animals were housed singly from the time of weaning (21 d) and maintained in a reverse light-dark housing room (lights on 21:00 and lights off 09:00) in standard housing cages with ad libitum food and water. Procedures were conducted in accordance with the National Institutes of Health guidelines and approved by the NIH Institutional Animal Care and Use Committee.

### Electroconvulsive Shock (ECS)

ECS was delivered to mice under isoflurane inhalation anesthesia via bilateral ear clip electrodes using an Applegate Electronics ES3 unit. The stimulus current was 60 mA, 60 Hz, sine wave of 1 s duration. The presence of tonic seizures immediately after the shock was confirmed by observing the extension of all limbs and forward head extension that normally last for about 10-15 s in each cohort regardless of genotype. Mice were returned to their cages 10 min following the procedure.

### Sleep Deprivation

Total sleep deprivation (12 h) was carried out according to previously described protocols [3,56]. Animals were gently handled, frequently exposed to novel objects and periodically changed to new cages. A single experimenter observed and handled the animals during the 12 h period. Mice were sleep deprived starting at 21:00 h and were continuously monitored throughout the ensuing light period. Mice in the control groups remained in their standard home caging.

### HomeCage Monitoring

Sleep behavior was analyzed using automated home cage monitoring for up to 48 h. Videos were captured within a sound-attenuated, temperature controlled environment with a constant white noise background designed by KM and RJS and built by CleverSys using digital cameras and CaptureStar software (CleverSys, Reston, VA). Infrared lights were used for illumination during dark phase recording. Automated video analysis of home cage behavior was performed using HomeCageScan software (CleverSys, Reston, VA). Behavior was detected by utilizing information about the entire body of the animal, identifying animal body parts such as head, tail, forelimbs, hind limbs, upper/lower back, abdomen, etc., and using the sequence data to automatically recognize and analyze animal behavior in durations > 6 frames (30 frames/s).

### RNA Extraction and cDNA conversion

Mice were quickly decapitated and brains rapidly removed from the skull. Brains were cut into 3mm coronal slices with the use of a brain block (Braintree Scientific, Braintree MA) and submerged in RNALater (Ambion). Slices remained in RNALater at 4°C for 3 d before microdissection of the mPFC. After microdissection, tissues pieces were transferred to Trizol (Invitrogen, Carlsbad, CA) and dounce homogenized. Following crude extraction, RNA was further purified on an RNeasy Column with on-column DNase treatment according to manufacturer's instructions (Qiagen, Valencia, CA). 0.5 ug of RNA was converted to cDNA using Superscript III (Invitrogen) according to the manufacturer's instructions.

### Quantitative PCR

10ng of reverse transcribed cDNA was subsequently used for each reaction with MGB FAM labeled TaqMan probes (Applied Biosystems, Foster City, CA) in 1X Gene Expression Master Mix (Applied Biosystems). Sequences for exon-specific *Bdnf *probes were adapted from previously published reports and ordered as custom TaqMan probes [57] (Applied Biosystems). Other genes (*Gad1, Calb1, Calb2, Pvalb, Sst, Npy, Cort, Tac1, Crhbp *and *Gapdh*) were ordered as inventoried TaqMan probes (Applied Biosystems). Each reaction was carried out in triplicate on a 4S Realplex Mastercycler (Eppendorf, Hamburg, Germany). PCR was carried out for 40 cycles of 95°C for 15 s and 60°C for 60 s. Relative quantification of template was performed using the ΔΔCt method with experimental cDNA data being normalized to the control *Gapdh *level. Absolute quantifications for the ratios of of individual transcript abundance was performed by extrapolating Ct value data to a standard curve, which was derived from serial dilution amplifications with known amounts of plasmid DNA containing the cloned sequence from PCR amplicons of individual *Bdnf *transcripts.

### BDNF ELISA

Mice were quickly decapitated and brains rapidly removed from the skull. Brains were cut into 3mm coronal slices with the use of a brain block and the mPFC was further dissected with the aid of a dissecting microscope. Tissue pieces were snap-frozen in an isopentane/dry-ice bath and frozen at -80°C until processing. Tissue pieces were dounced in lysis buffer (150mM NaCl, 10mM Tris-Cl pH 7.2, 0.1% SDS, 1% Triton X-100, 1% Deoxycholate and 5 mM EDTA), sonicated and further extracted for 1 h with addition of 0.9% SDS. Protein levels were normalized using a standard BCA assay (Pierce) and then used for measurement of BDNF protein levels with a commercial BDNF ELISA according to the manufacturer's instructions (Millipore, Billerica, MA).

### Statistics

GraphPad Prism was used for all statistics including Students t-test, one-way ANOVA with Newman-Keuls post hoc analysis and two-way ANOVA. *p < 0.05, **p < 0.01 and ***p < 0.001.

## List of abbreviations used

BDNF: Brain-derived neurotrophic factor; CRH: corticotropin releasing hormone; ECS: electroconvulsive shock; EEG: electroencephalogram; GFP: green fluorescent protein; mPFC: medial prefrontal cortex; PGK: phosphoglycerate kinase; SD: sleep deprivation; SWA: Slow-wave activity; WT: wild-type.

## Competing interests

KM, RJS, DVJ and DRW declare no competing financial or non-financial competing interests. BL is a paid employee of Glaxo-Smith Kline.

## Authors' contributions

KM conceived the study, designed the experiments, carried out the qPCR and immunoassay studies and drafted the manuscript. RJS participated in designing and coordinating experiments and carried out qPCR experiments. DVJ carried out behavioral experiments. DRW contributed to study design and analysis. BL contributed to study design, analysis and drafted the manuscript. All authors have read and approved the final manuscript.

## Supplementary Material

Additional file 1 **Additional file 1**. Table S1: "ANOVA table" Click here for file
