# Supplementary material for: Activity-dependent brain-derived neurotrophic factor expression regulates cortistatin-interneurons and sleep behavior
Source: Mol Brain. 2011 Mar 9;4:11. doi: 10.1186/1756-6606-4-11 (PMC3061911; doi:10.1186/1756-6606-4-11)
Supplement: Additional file 1 — Table S1: "ANOVA table" [file 1756-6606-4-11-S1.PDF]

Figure 1A	Test	Main Effect (genotype)	Main Effect (ECS)	Interaction (genotype X ECS)
Exon I	Two-way ANOVA	F(1,8)=56.44, p<0.0001	F(1,8)=19.18, p=0.0023	F(1,8)=4.51, p=0.0664
Exon II	Two-way ANOVA	F(1,8)=224.28, p<0.0001	F(1,8)=13.32, p=0.0065	F(1,8)=4.81, p=0.0596
Exon III	Two-way ANOVA	F(1,8)=137.76, p<0.0001	F(1,8)=25.79, p=0.001	F(1,8)=7.37, p=0.0264
Exon IV	Students t-test	N/A	F(2,2)=69.99, p=0.0366	N/A
Exon V	Two-way ANOVA	F(1,8)=4.95, p=0.0568	F(1,8)=0.30, p=0.5986	F(1,8)=0.86, p=0.3816
Exon VI	Two-way ANOVA	F(1,8)=20.62, p=0.0019	F(1,8)=140.74, p<0.0001	F(1,8)=1.57, p=0.2458
Exon VIII	Two-way ANOVA	F(1,8)=0.95, p=0.3590	F(1,8)=1.74, P=0.2240	F(1,8)=0.01, p=0.9073
Exon IXa	Two-way ANOVA	F(1,8)=52.84, p<0.0001	F(1,8)=23.51, p=0.0013	F(1,8)=20.03, p=0.0021
Figure 1B	Test	Main Effect (genotype)	Main Effect (Time of Day)	Interaction (genotype X Time of Day)
Exon I	Two-way ANOVA	F(1,8)=19.92, p=0.0021	F(1,8)=11.17, p=0.0102	F(1,8)=4.13, p=0.0766
Exon II	Two-way ANOVA	F(1,8)=32.93, p=0.0004	F(1,8)=6.35, p=0.0358	F(1,8)=0.84, p=0.3853
Exon III	Two-way ANOVA	F(1,8)=406.37, p=<0.0001	F(1,8)=5.56, p=0.0462	F(1,8)=4.23, p=0.0736
Exon IV	Students t-test	N/A	F(2,2)=1.688, p=0.0331	N/A
Exon V	Two-way ANOVA	F(1,8)=24.77, p=0.0011	F(1,8)=1.52, p=0.2530	F(1,8)=1.72, p=0.2259
Exon VI	Two-way ANOVA	F(1,8)=17.91, p=0.0029	F(1,8)=4.26, p=0.0729	F(1,8)=0.35, p=0.5682
Exon VIII	Two-way ANOVA	F(1,8)=2.88, p=0.1278	F(1,8)=0.88, p=0.3768	F(1,8)=0.64, p=0.4465
Exon Ixa	Two-way ANOVA	F(1,8)=11.85, p=0.0088	F(1,8)=10.76, p=0.0112	F(1,8)=2.77, p=0.1347
Figure 2A	Test	Main Effect (genotype)	Main Effect (Sleep Deprivation)	Interaction (genotype X Sleep Deprivation)
Exon I	Two-way ANOVA	F(1,8)=170.56, p<0.0001	F(1,8)=247.69, p<0.0001	F(1,8)=127.06, p<0.0001
Exon II	Two-way ANOVA	F(1,8)=561.80, p<0.0001	F(1,8)=231.32, p<0.0001	F(1,8)=99.52, p<0.0001
Exon III	Two-way ANOVA	F(1,8)=71.37, p<0.0001	F(1,8)=13.71, p=0.006	F(1,8)=7.83, p=0.0233
Exon IV	Students t-test	N/A	F(2,2)=72.26, p=0.0018	N/A
Exon V	Two-way ANOVA	F(1,8)=0.44, p=0.5237	F(1,8)=1.09, p=0.3276	F(1,8)=0.11, p=0.7529
Exon VI	Two-way ANOVA	F(1,8)=1.69, p=0.2302	F(1,8)=28.57, p=0.0007	F(1,8)=5.57, p=0.0459
Exon VIII	Two-way ANOVA	F(1,8)=3.42, p=0.1018	F(1,8)=1.02, p=0.3413	F(1,8)=0.11, p=0.7445
Exon Ixa	Two-way ANOVA	F(1,8)=36.64, p=0.0003	F(1,8)=24.09, p=0.0012	F(1,8)=16.19, p=0.0038
Figure 2B		Main Effect (genotype)	Main Effect (Sleep Deprivation)	Interaction (genotype X Sleep Deprivation)
	Two-way ANOVA	F(1,12)=17.17, p=0.0014	F(1,12)=9.98, p=0.0082	F(1,12)=6.01, p=0.0305
Figure 4	Test	Main Effect (genotype)	Main Effect (Sleep Deprivation)	Interaction (genotype X Sleep Deprivation)
Gad1	Two-way ANOVA	F(1,8)=0.47, p=0.5133	F(1,8)=3.41, p=0.1021	F(1,8)=0.64, p=0.4456
Pvalb	Two-way ANOVA	F(1,8)=0.50, p=0.499	F(1,8)=0.1, p=0.7612	F(1,8)=0.22, p=0.6539
Calb1	Two-way ANOVA	F(1,8)=0.65, p=0.4418	F(1,8)=4.06, p=0.0786	F(1,8)=0.38, p=0.5571
Calb2	Two-way ANOVA	F(1,8)=10.88, p=0.0109	F(1,8)=0.1, p=0.7589	F(1,8)=0.08, p=0.7785
Figure 5				
Npy	Two-way ANOVA	F(1,8)=14.54,p=0.0051	F(1,8)=3.75, p=0.0887	F(1,8)=0.12, p=0.7345
Sst	Two-way ANOVA	F(1,8)=28.79, p=0.0007	F(1,8)=3.33, p=0.1055	F(1,8)=1.16, p=0.3129
Cort	Two-way ANOVA	F(1,8)=143.14, p<0.0001	F(1,8)=49.22, p=0.0001	F(1,8)=14.17, p=0.0055
Tac1	Two-way ANOVA	F(1,8)=98.24, p<0.0001	F(1,8)=31.38, p=0.0005	F(1,8)=2.62, p=0.1439
Crhbp	Two-way ANOVA	F(1,8)=118.35, p<0.0001	F(1,8)=39.75, p=0.0002	F(1,8)=9.77, p=0.0141
